# Supplementary figures and images for: Maximal interferon induction by influenza lacking NS1 is infrequent owing to requirements for replication and export
Source: PLoS Pathog. 2023 Apr 17;19(4):e1010943. doi: 10.1371/journal.ppat.1010943 (PMC10138204; doi:10.1371/journal.ppat.1010943)

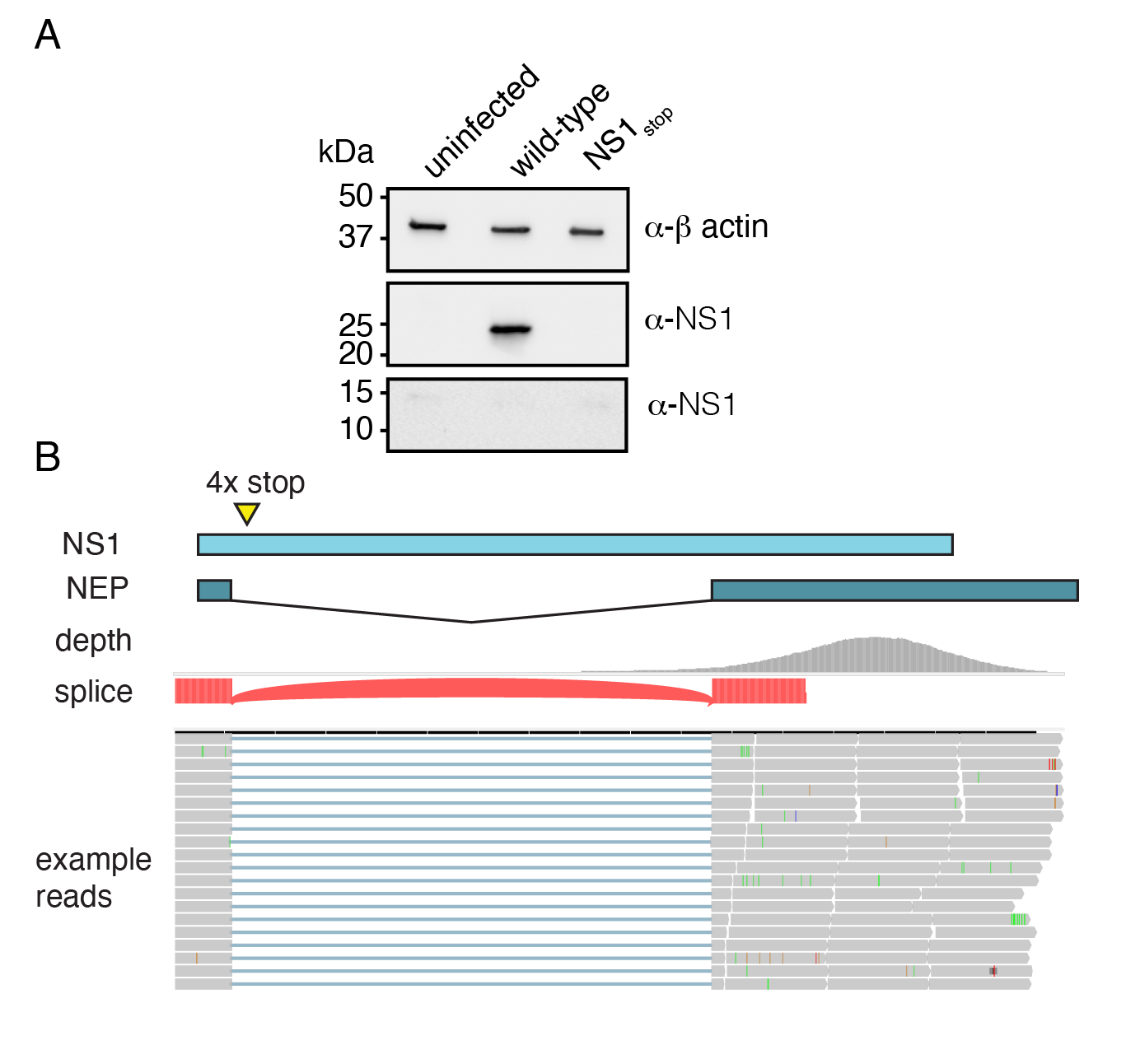

Supplement: S1 Fig — (A) Western blot analysis of indicated viral infections at an MOI of 5 in A549 cells at 24hpi using the indicated antibodies. NS1stop mutation leads to non-detectible NS1 expression (middle, expected size 26 kDa). Our NS1 antibody does not produce a strong band consistent in size with NEP (bottom, expected size 14.3 kDa), indicating it is largely, if not solely, specific to NS1. Equal loading across lanes (top, α-β-actin, expected size, 43 kDa). (B) NS1stop retains splicing. Schematic of NS1stop mutant alongside IGV sequencing visualization of data described in S3 Fig. [74] Splice site identified by sequencing matches the canonical NEP splice site. Example reads supporting splice site shown. Splice site excludes stop codons introduced into NS1stop. (TIF) [file ppat.1010943.s013.tif]

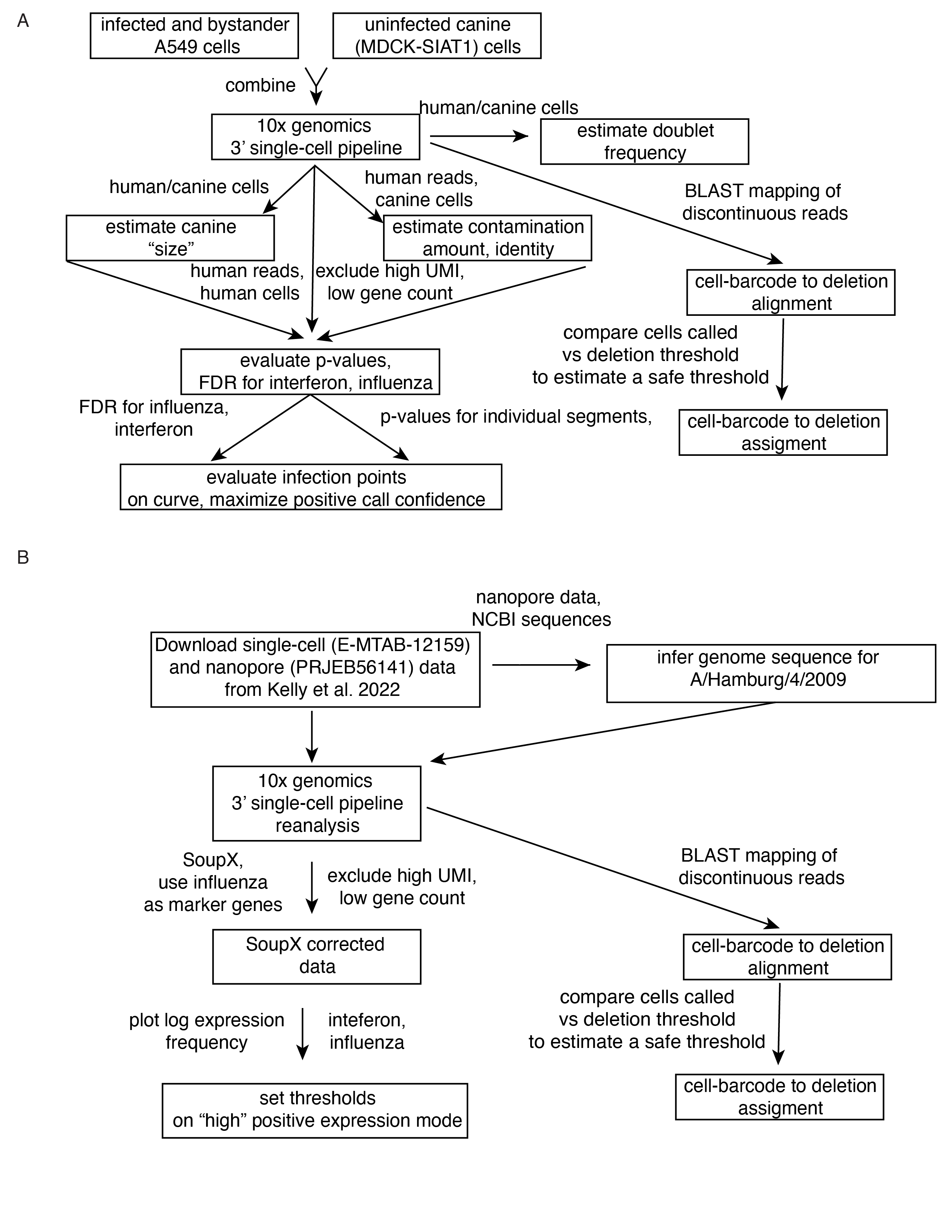

Supplement: S2 Fig — Processing schematic for WSN data generated in this study on A549 cells (A) and reprocessing of Kelly et al. data of A/Hamburg/4/2009 in NHBE cells (B). Further description in methods and in accompanying github repository (https://github.com/Russell-laboratory/NS1_interferon_variation). (TIF) [file ppat.1010943.s014.tif]

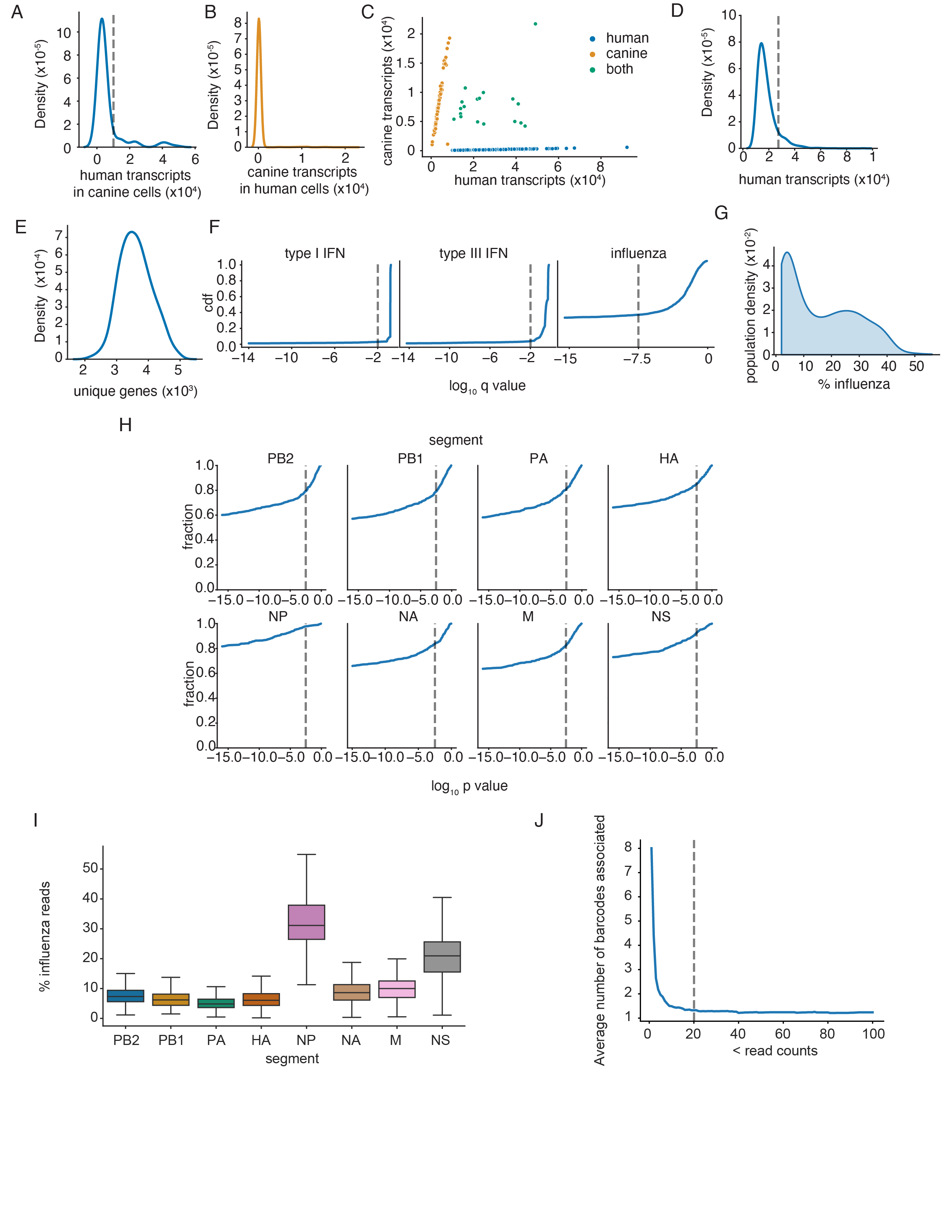

Supplement: S3 Fig — Thresholding for human cells based on contaminating fractions in canine cells for NS1stop, and deletion thresholding. (A) Distribution of human transcripts found in cells identified as canine by Cellranger software. We found misannotation of some doublets, which we reannotated, denoted by the dotted line. (B) Distribution of canine transcripts found in cells identified as human by Cellranger. No signs of misannotation as in A. (C) Final droplet annotations after modification. (D) Distribution of transcript counts in droplets annotated as containing only human cells. To try and exclude doublets from further analysis, droplets with content greater than the dotted line were excluded. (E) Distribution of unique genes with >0 counts in thresholded human cells. As distribution was normal, no high or low counts needed to be excluded. (F) Using inferred contamination from A and C, assuming a constant fraction of reads contaminating droplets, with a correction for the relative number of transcripts recovered from human versus canine droplets, q values were interpolated from p-values assuming a Poisson sampling and Benjamini-Hochberg multiple testing correction. Empirically-derived thresholds were derived, as shown by the dotted line, at inflection points for interferons and at a reasonably conservative estimate for influenza. Data show the cumulative density function (cdf), the fraction of droplets (of the total) that meet the indicated threshold. (G) Distribution of influenza transcript frequencies (as a percentage of all transcripts recovered from a cell) in influenza-positive cells. (H) The fraction of influenza positive cells that meet the indicated p-value threshold for presence of the indicated segment from our calculations using contamination measured by canine cells. P-values rather than q-values were used as both presence and absence are of interest, and we have pre-conditioned on influenza-positive cells alone. Dotted lines indicate p-value thresholds for positivit [file ppat.1010943.s015.tif]

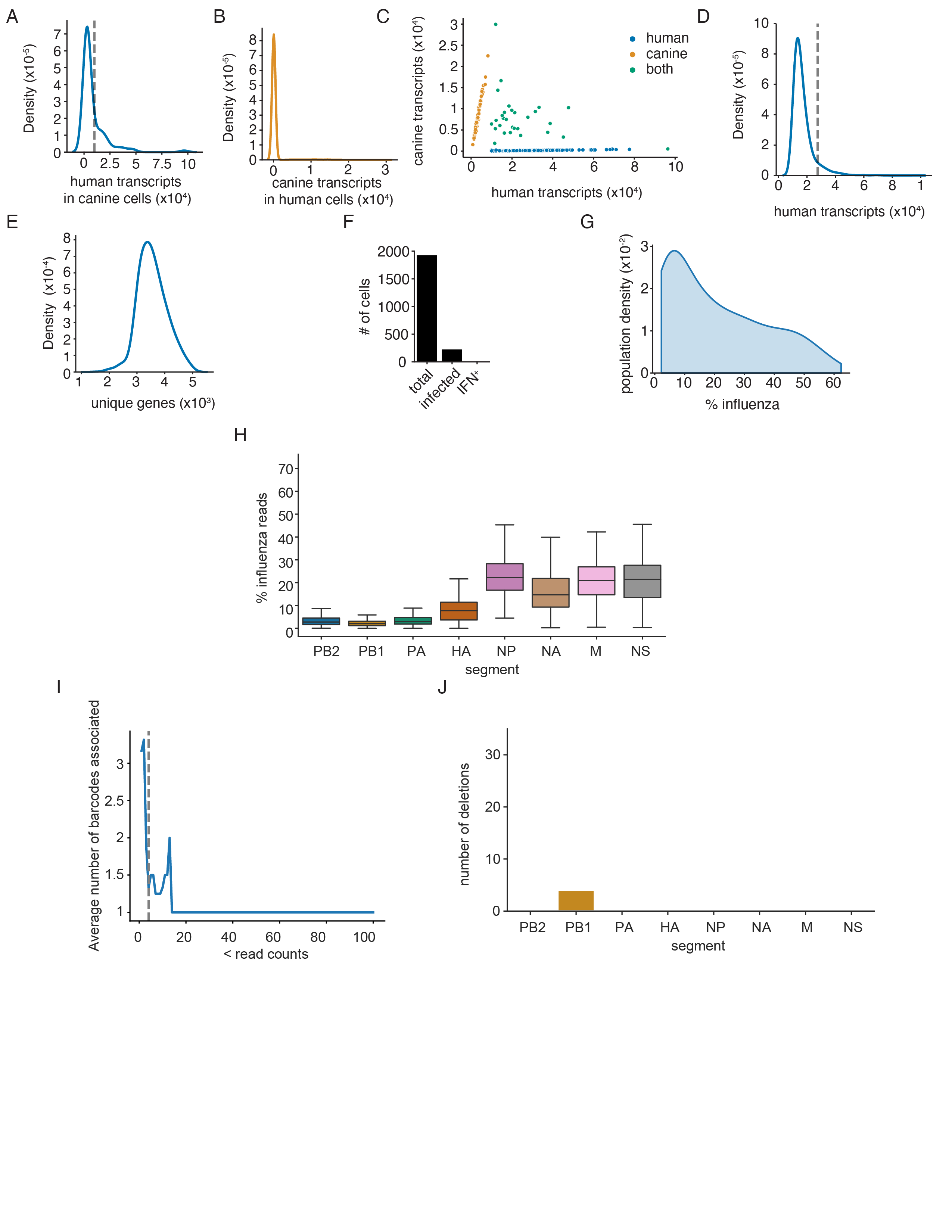

Supplement: S4 Fig — Thresholding for human cells based on contaminating fractions in canine cells for wild-type WSN infection, and deletion thresholding. Infections were performed at an MOI of 0.2 and harvested at 10hpi, as in S3 Fig. (A) Distribution of human transcripts found in cells identified as canine by Cellranger software. We found misannotation of some doublets, which we reannotated, denoted by the dotted line. (B) Distribution of canine transcripts found in cells identified as human by Cellranger. No signs of misannotation as in A. (C) Final droplet annotations after modification. (D) Distribution of transcript counts in droplets annotated as containing only human cells. To try and exclude doublets from further analysis, droplets with content greater than the dotted line were excluded. (E) Distribution of unique genes with >0 counts in thresholded human cells. As distribution was normal, no high or low counts needed to be excluded. (F) Summary of number of cells called in each category. (G) Distribution of influenza transcript frequencies (as a percentage of all transcripts recovered from a cell) in influenza-positive cells. (H) Fraction of influenza reads derived from each influenza segment in influenza-positive cells. (I) The number of average emulsions associated with any given deletion at the indicated read support is shown. At lower read support, deletions are more broadly distributed, suggesting contamination or template-switching. At higher support, they are less broadly distributed, suggesting bone-fide deletions. Cutoff chosen for this study indicated by the dotted line. (J) Deletion identity and counts from I. (TIF) [file ppat.1010943.s016.tif]

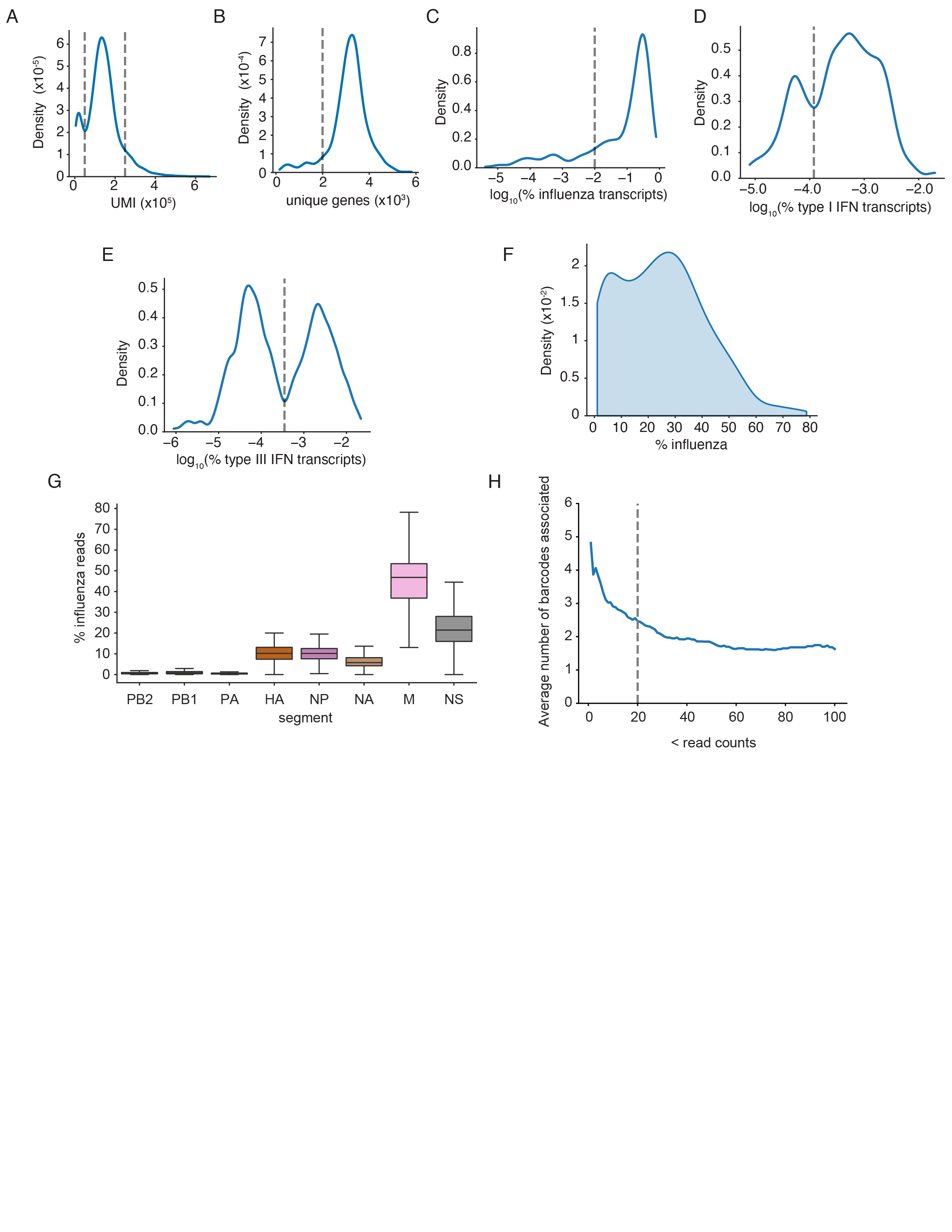

Supplement: S5 Fig — Thresholding after SoupX to identify confident-positive populations. (A) Low and high UMI cells were excluded as poor-quality or doublets (dotted lines). (B) Distribution of unique genes with >0 counts in thresholded human cells. Cells with less than 2000 unique, non-zero, genes were excluded from this analysis as poor-quality (dotted line). (C) Density distribution of the log fraction of transcripts derived from influenza in all cells with at least one influenza-derived UMI after SoupX correction. Threshold was set to include the two highest modes, but exclude lower modes consistent with only one or two reads derived from influenza (dotted line). (D) Density distribution of the log fraction of transcripts derived from type I interferons in all cells with at least one type I interferon-derived UMI after SoupX correction. Threshold was set to include the highest mode, but exclude lower mode consistent with only one or two reads derived from a type I interferon (dotted line). (E) Density distribution of the log fraction of transcripts derived from type III interferons in all cells with at least one type III interferon-derived UMI after SoupX correction. Threshold was set to include the highest mode, but exclude lower mode consistent with only one or two reads derived from a type III interferon (dotted line). (F) Distribution of influenza transcript frequencies (as a percentage of all transcripts recovered from a cell) in influenza-positive cells. (G) Fraction of influenza reads derived from each influenza segment in influenza-positive cells. (H) The number of average emulsions associated with any given deletion at the indicated read support is shown. At lower read support, deletions are more broadly distributed, suggesting contamination or template-switching. At higher support, they are less broadly distributed, suggesting bone-fide deletions. Cutoff chosen for this study indicated by the dotted line. (TIF) [file ppat.1010943.s017.tif]

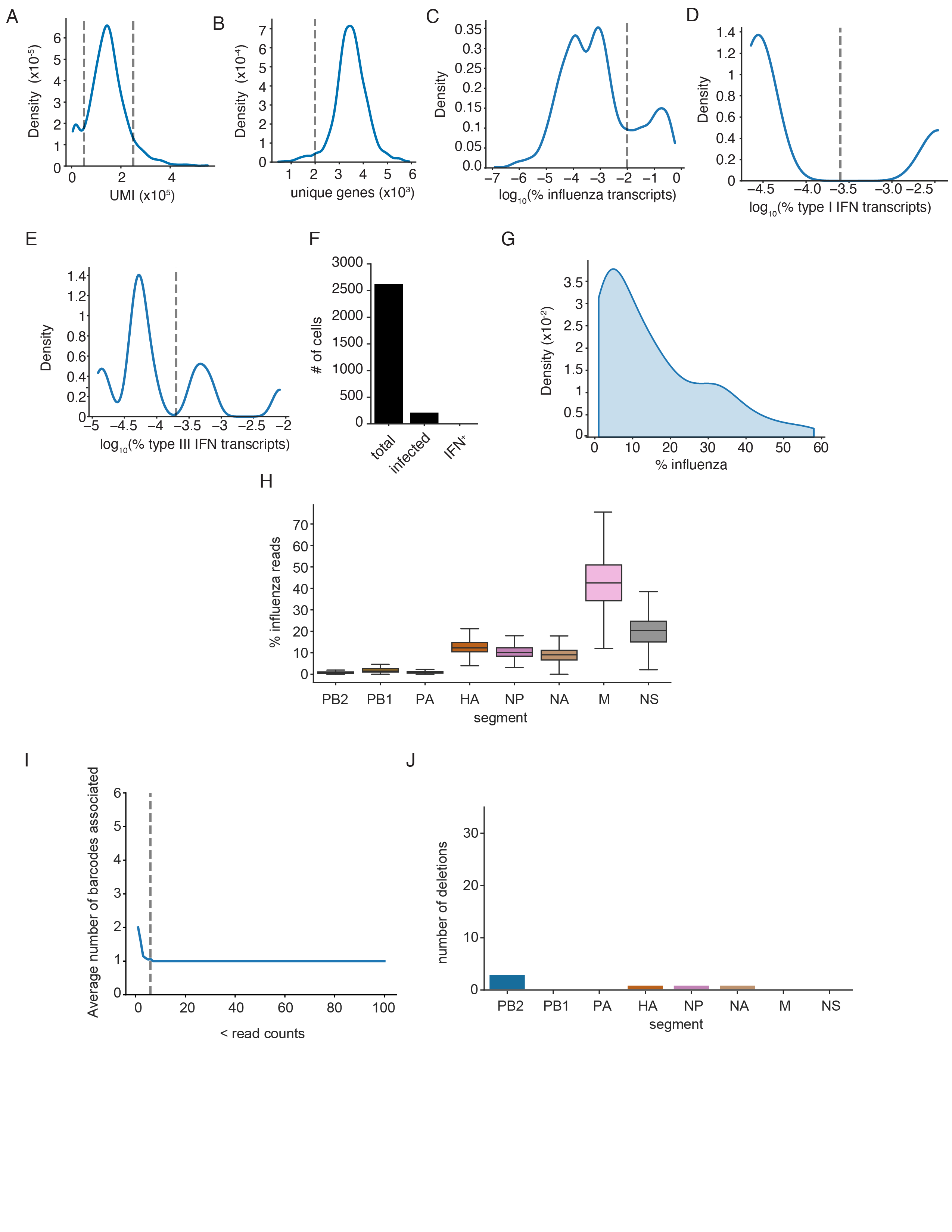

Supplement: S6 Fig — Thresholding after SoupX to identify confident-positive populations. (A) Low and high UMI cells were excluded as poor-quality or doublets (dotted lines). (B) Distribution of unique genes with >0 counts in thresholded human cells. Cells with less than 2000 unique, non-zero, genes were excluded from this analysis as poor-quality (dotted line). (C) Density distribution of the log fraction of transcripts derived from influenza in all cells with at least one influenza-derived UMI after SoupX correction. Threshold was set to be consistent with that set in S5 Fig. (D) Density distribution of the log fraction of transcripts derived from type I interferons in all cells with at least one type I interferon-derived UMI after SoupX correction. Threshold was set to include the highest mode, but exclude lower mode consistent with only one or two reads derived from a type I interferon (dotted line). (E) Density distribution of the log fraction of transcripts derived from type III interferons in all cells with at least one type III interferon-derived UMI after SoupX correction. Threshold was set to include the highest mode, but exclude lower mode consistent with only one or two reads derived from a type III interferon (dotted line). (F) Summary of cells after thresholding. There were five interferon-positive cells. (G) Distribution of influenza transcript frequencies (as a percentage of all transcripts recovered from a cell) in influenza-positive cells. (H) Fraction of influenza reads derived from each influenza segment in influenza-positive cells. (I) The number of average emulsions associated with any given deletion at the indicated read support is shown. At lower read support, deletions are more broadly distributed, suggesting contamination or template-switching. At higher support, they are less broadly distributed, suggesting bone-fide deletions. Cutoff chosen for this study indicated by the dotted line. (J) Summary of deletion counts after thresholding in I. (TIF) [file ppat.1010943.s018.tif]

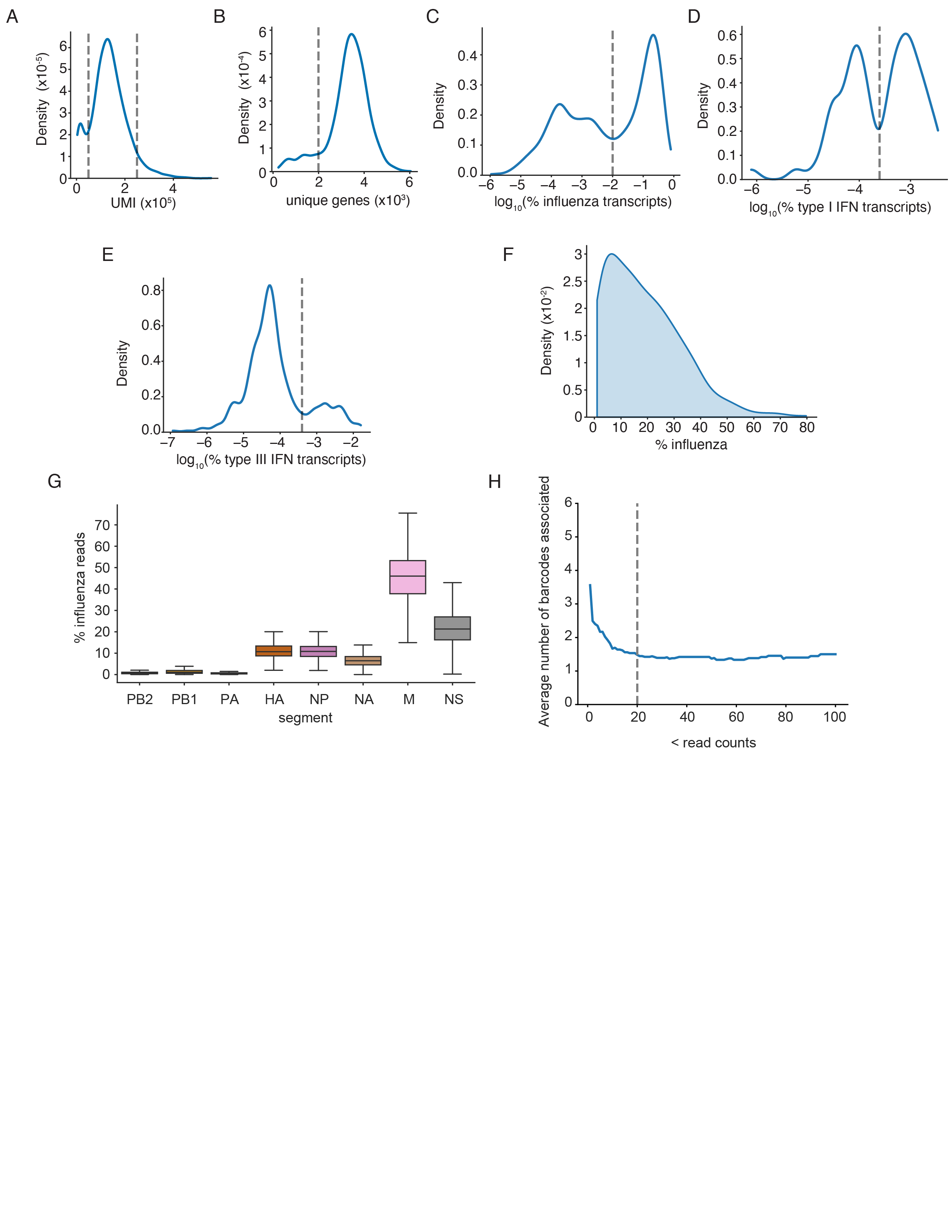

Supplement: S7 Fig — Thresholding after SoupX to identify confident-positive populations. (A) Low and high UMI cells were excluded as poor-quality or doublets (dotted lines). (B) Distribution of unique genes with >0 counts in thresholded human cells. Cells with less than 2000 unique, non-zero, genes were excluded from this analysis as poor-quality (dotted line). (C) Density distribution of the log fraction of transcripts derived from influenza in all cells with at least one influenza-derived UMI after SoupX correction. Threshold was set to include the highest mode, but exclude lower modes consistent with only one or two reads derived from influenza (dotted line). (D) Density distribution of the log fraction of transcripts derived from type I interferons in all cells with at least one type I interferon-derived UMI after SoupX correction. Threshold was set to include the highest mode, but exclude lower mode consistent with only one or two reads derived from a type I interferon (dotted line). (E) Density distribution of the log fraction of transcripts derived from type III interferons in all cells with at least one type III interferon-derived UMI after SoupX correction. Threshold was set to include the highest mode, but exclude lower mode consistent with only one or two reads derived from a type III interferon (dotted line). (F) Distribution of influenza transcript frequencies (as a percentage of all transcripts recovered from a cell) in influenza-positive cells. (G) Fraction of influenza reads derived from each influenza segment in influenza-positive cells. (H) The number of average emulsions associated with any given deletion at the indicated read support is shown. At lower read support, deletions are more broadly distributed, suggesting contamination or template-switching. At higher support, they are less broadly distributed, suggesting bone-fide deletions. Cutoff chosen for this study indicated by the dotted line. (TIF) [file ppat.1010943.s019.tif]

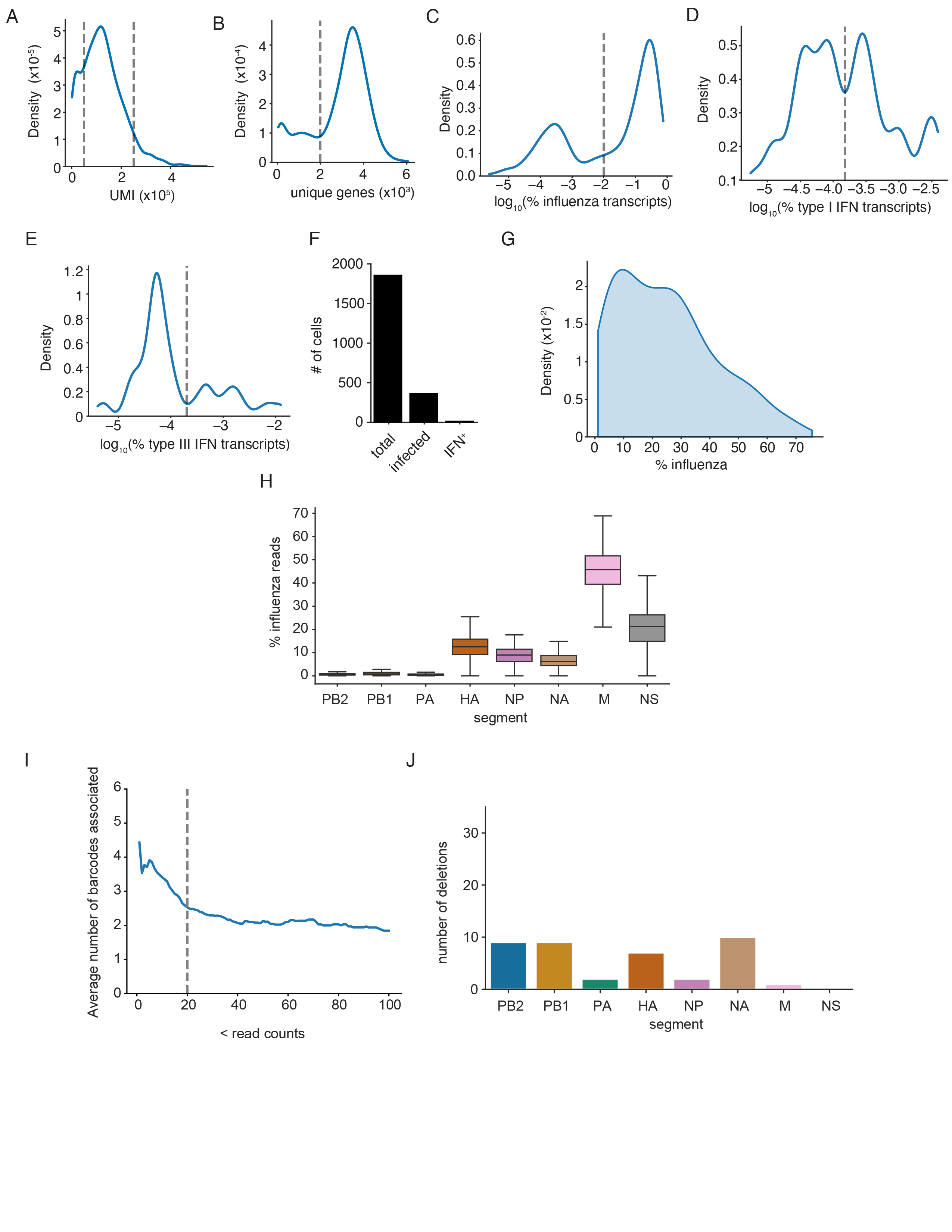

Supplement: S8 Fig — Thresholding after SoupX to identify confident-positive populations. (A) Low and high UMI cells were excluded as poor-quality or doublets (dotted lines). (B) Distribution of unique genes with >0 counts in thresholded human cells. Cells with less than 2000 unique, non-zero, genes were excluded from this analysis as poor-quality (dotted line). (C) Density distribution of the log fraction of transcripts derived from influenza in all cells with at least one influenza-derived UMI after SoupX correction. Threshold was set to be consistent with that set in S5 Fig. (D) Density distribution of the log fraction of transcripts derived from type I interferons in all cells with at least one type I interferon-derived UMI after SoupX correction. Threshold was set to include the highest modes, but exclude lower mode consistent with only one or two reads derived from a type I interferon (dotted line). (E) Density distribution of the log fraction of transcripts derived from type III interferons in all cells with at least one type III interferon-derived UMI after SoupX correction. Threshold was set to include the highest modes, but exclude lower mode consistent with only one or two reads derived from a type III interferon (dotted line). (F) Summary of cells after thresholding. (G) Distribution of influenza transcript frequencies (as a percentage of all transcripts recovered from a cell) in influenza-positive cells. (H) Fraction of influenza reads derived from each influenza segment in influenza-positive cells. (I) The number of average emulsions associated with any given deletion at the indicated read support is shown. At lower read support, deletions are more broadly distributed, suggesting contamination or template-switching. At higher support, they are less broadly distributed, suggesting bone-fide deletions. Cutoff chosen for this study indicated by the dotted line. (J) Summary of deletion counts after thresholding in I. (TIF) [file ppat.1010943.s020.tif]

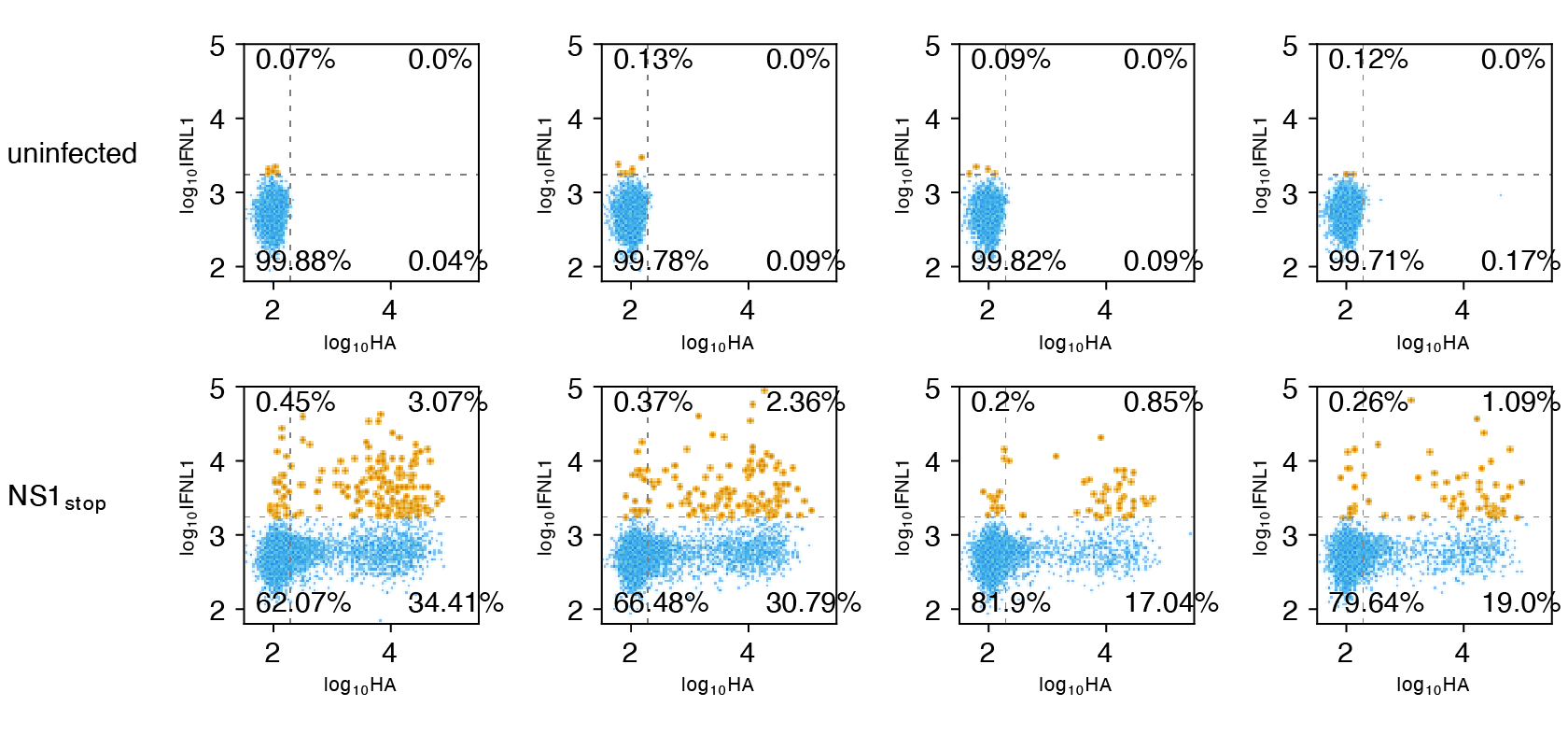

Supplement: S9 Fig — Data showing full flow data and measurements of IFNL1 reporter, and HA staining. Cells infected at an MOI of 0.1, measurements made 13h post-infection. Individual replicates shown. Interferon-positive events colored in orange. Data subsetted to 5000 events to show equivalent numbers between conditions. (TIF) [file ppat.1010943.s021.tif]

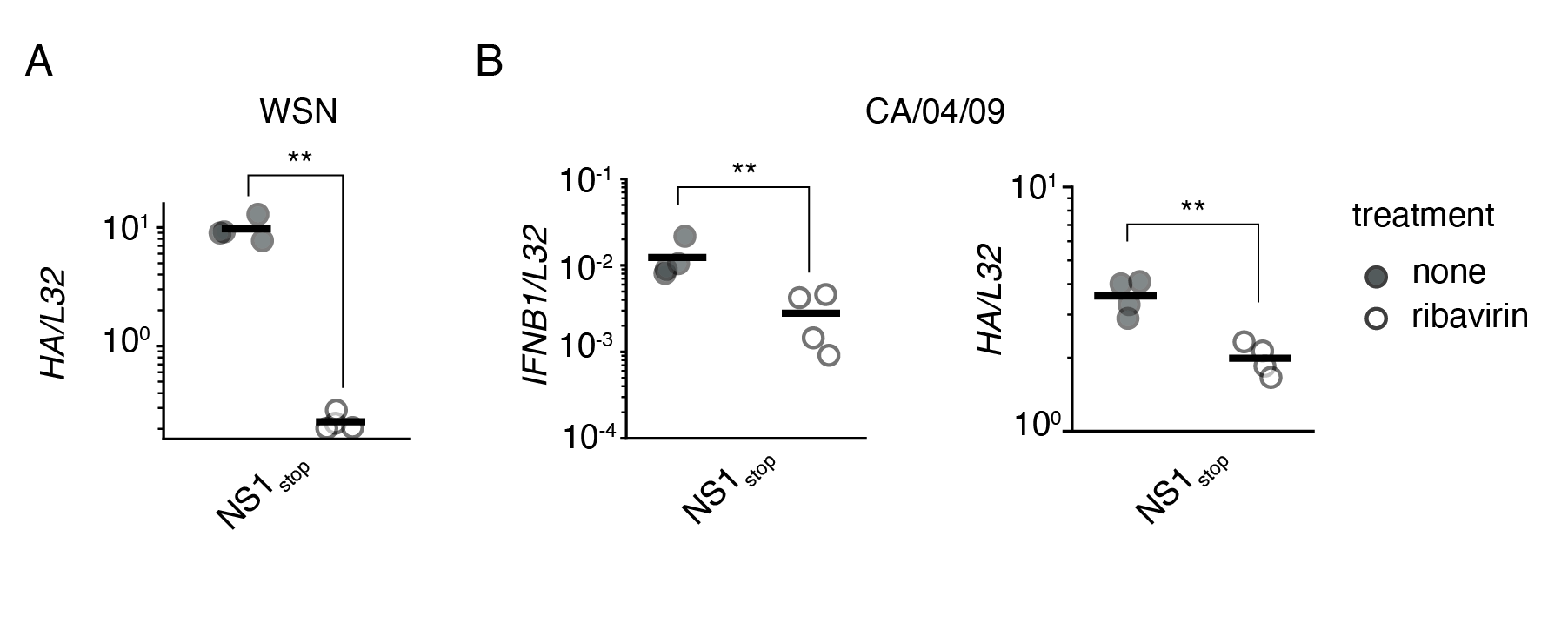

Supplement: S10 Fig — (A) Measurement of HA transcripts from experiment shown in Fig 2F. (B) A549 cells were treated for one hour with 200μM ribavirin prior to infection CA/04/2009 NS1stop at an MOI of 0.2. RNA was harvested at 9hpi, and analyzed by qPCR against IFNB1 (left) and HA (right) as compared to the housekeeping control, L32. Asterisks indicate significant difference, two-tailed t test, p<0.05, n = 4. (TIF) [file ppat.1010943.s022.tif]

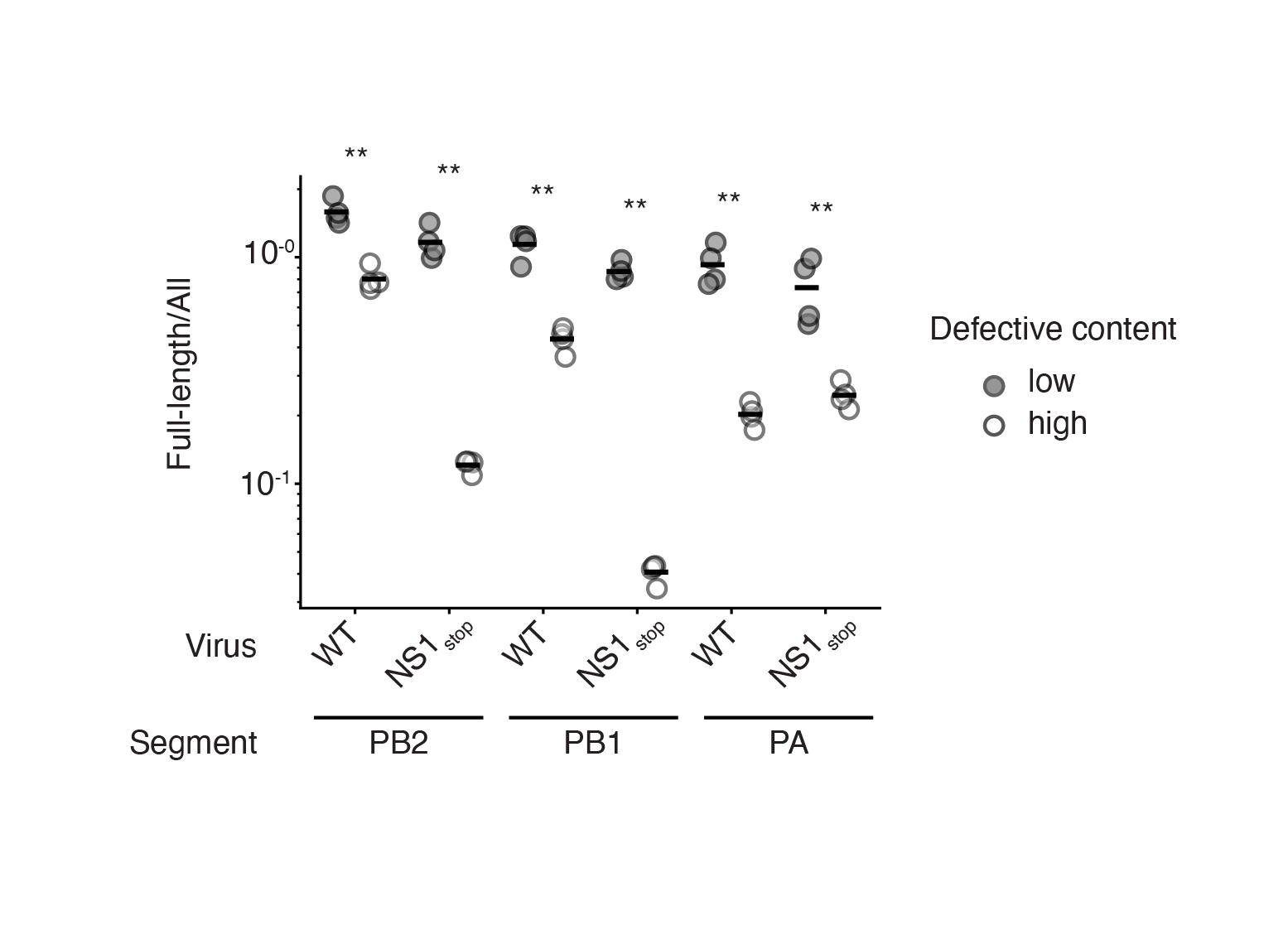

Supplement: S11 Fig — Indicated viral populations were subjected to influenza-specific cDNA synthesis and analyzed by a set of primers that either sit external to deletions (All) or internal (Full-length). The Full-length signal was normalized to the All signal for each segment, smaller numbers represent a depletion of full-length segment relative to the total population. Asterices indicate a significant depletion between high and low defective populations, two-tailed t test with Benjamini-Hochberg multiple testing correction at an FDR of 0.05, n = 4 two technical and two biological replicates. (TIF) [file ppat.1010943.s023.tif]

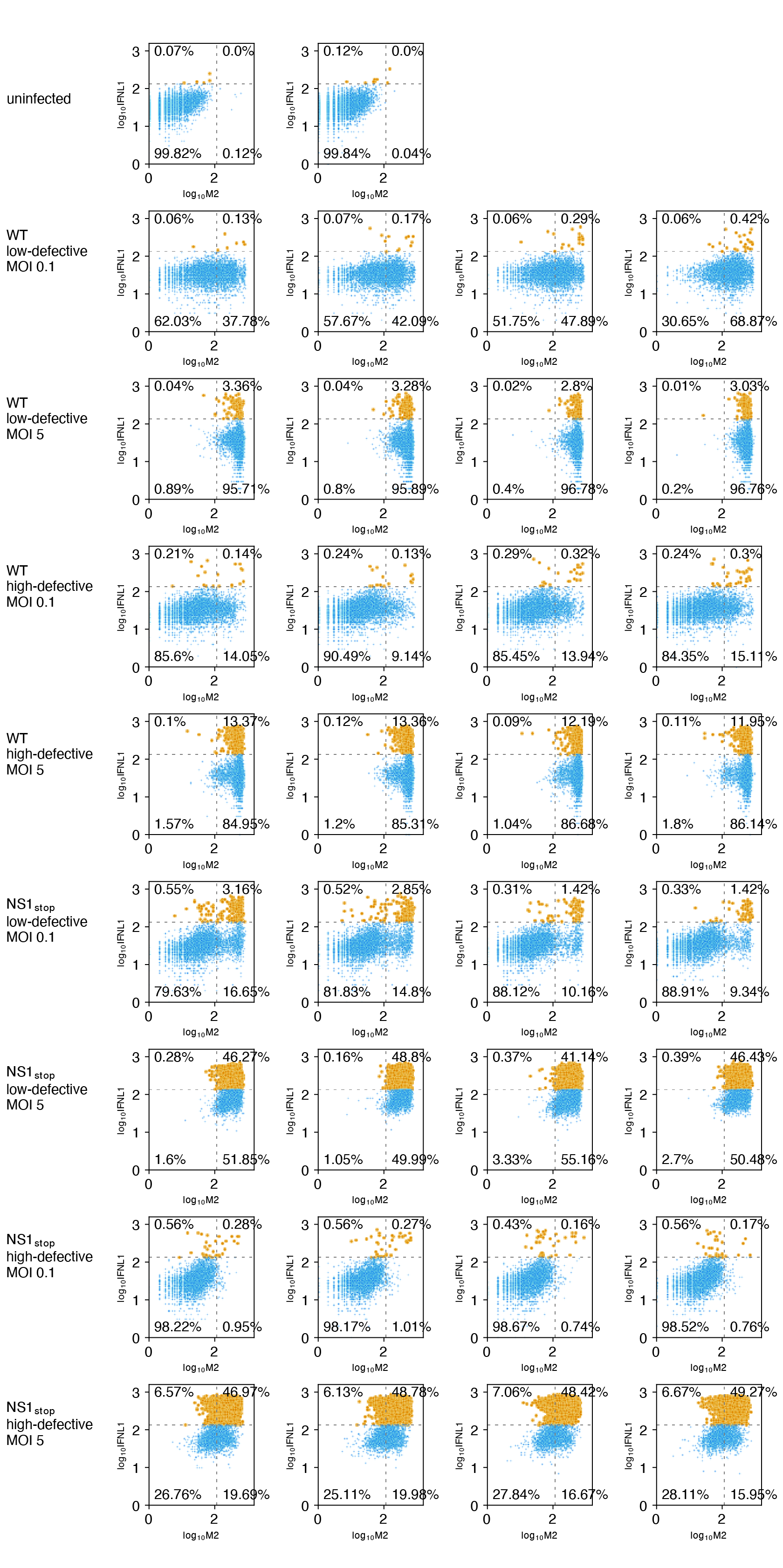

Supplement: S12 Fig — Data showing full flow data and measurements of IFNL1 reporter, and M2 staining. Cells infected the indicated, particle-corrected MOI, and measurements made 13h post-infection. Individual replicates shown. Interferon-positive events colored in orange. Data subsetted to 5000 events to show equivalent numbers between conditions. (TIF) [file ppat.1010943.s024.tif]

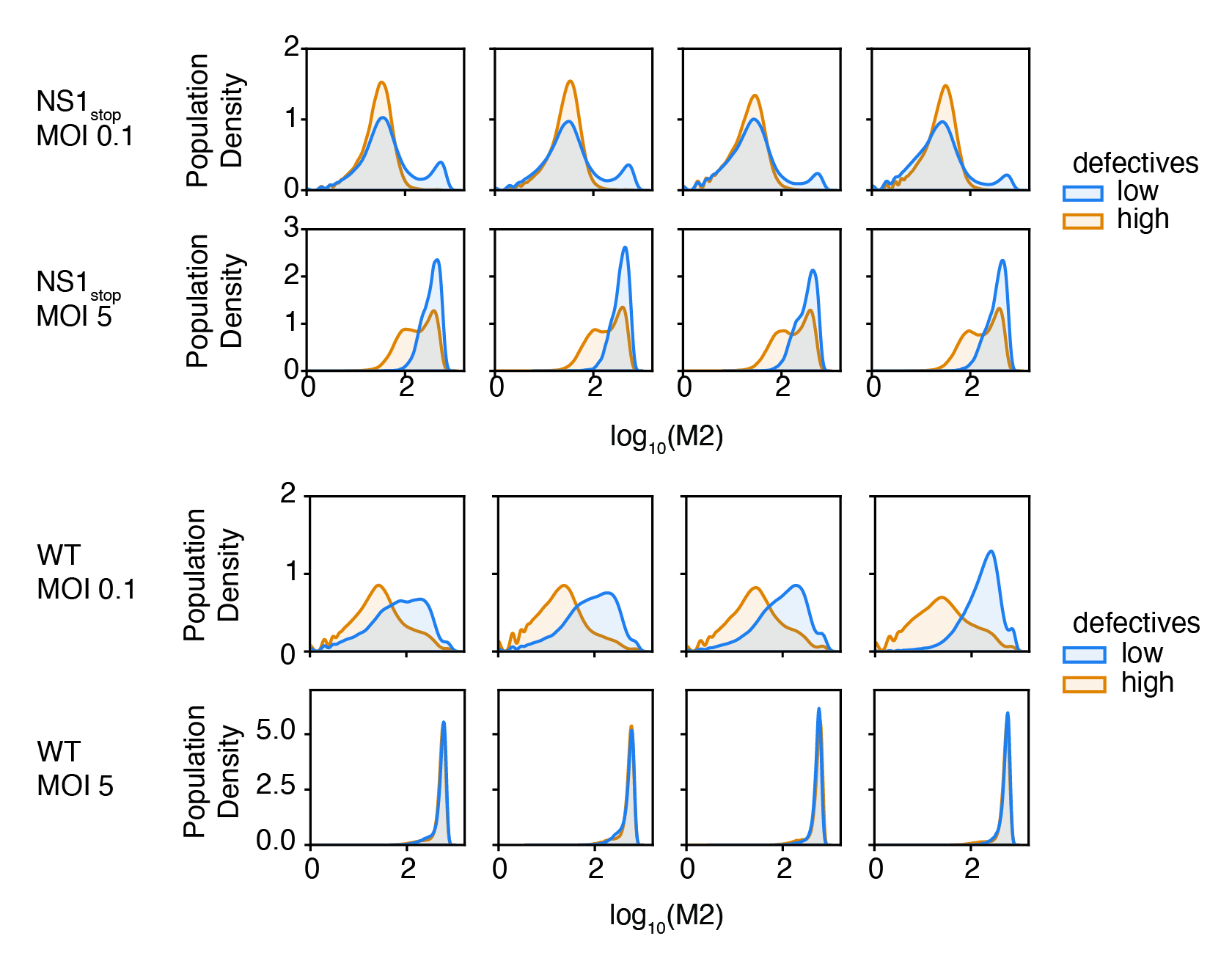

Supplement: S13 Fig — Density distributions for M2 staining from S12 Fig. (TIF) [file ppat.1010943.s025.tif]

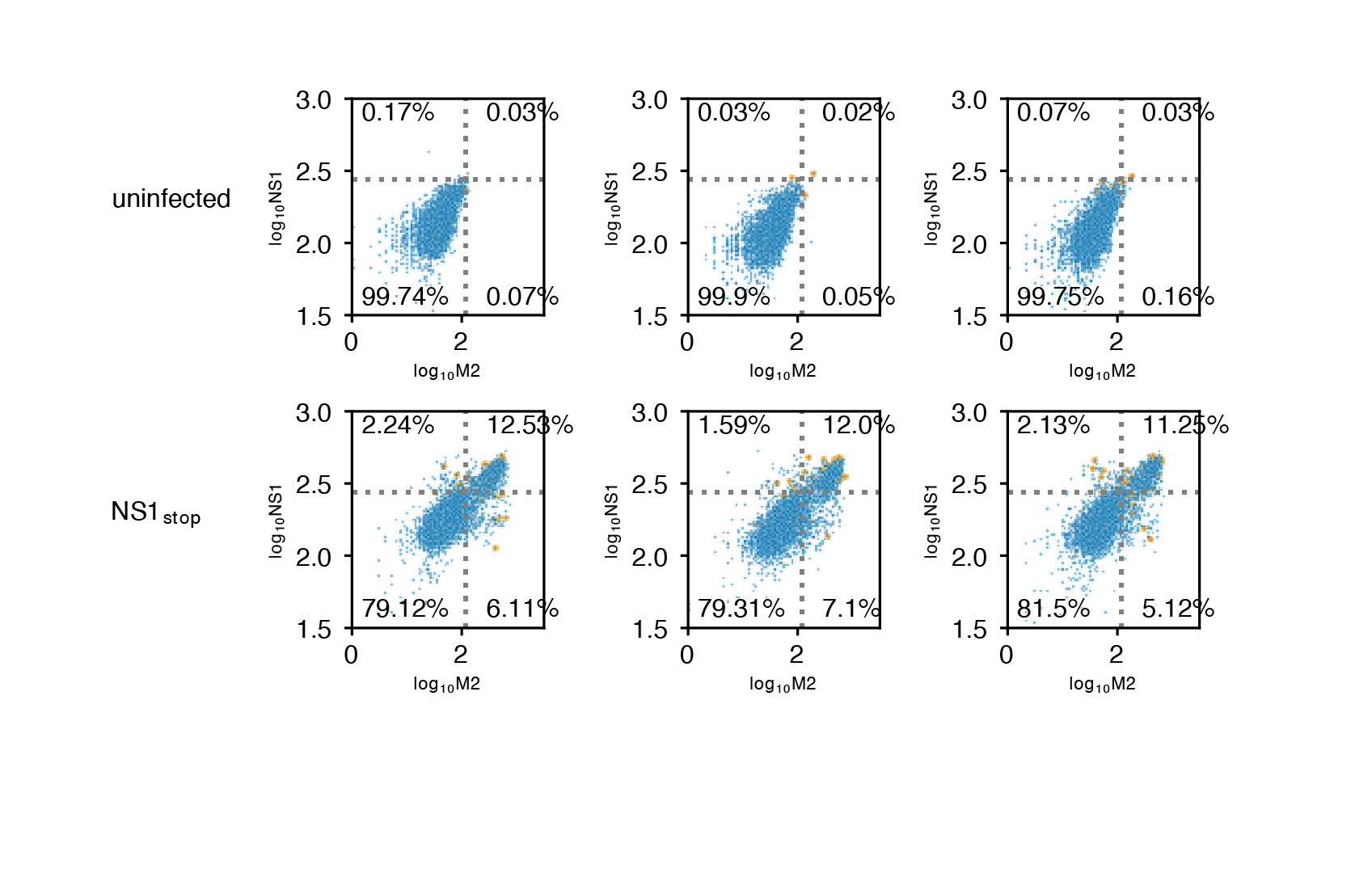

Supplement: S14 Fig — Data showing full flow data and measurements of IFNL1 reporter, M2, and NS1 staining of NS1stop variant. Cells infected at an MOI of 0.1, measurements made 13h post-infection. Individual replicates shown. Interferon-positive events colored in orange. Data subsetted to 5000 events to show equivalent numbers between conditions. (TIF) [file ppat.1010943.s026.tif]

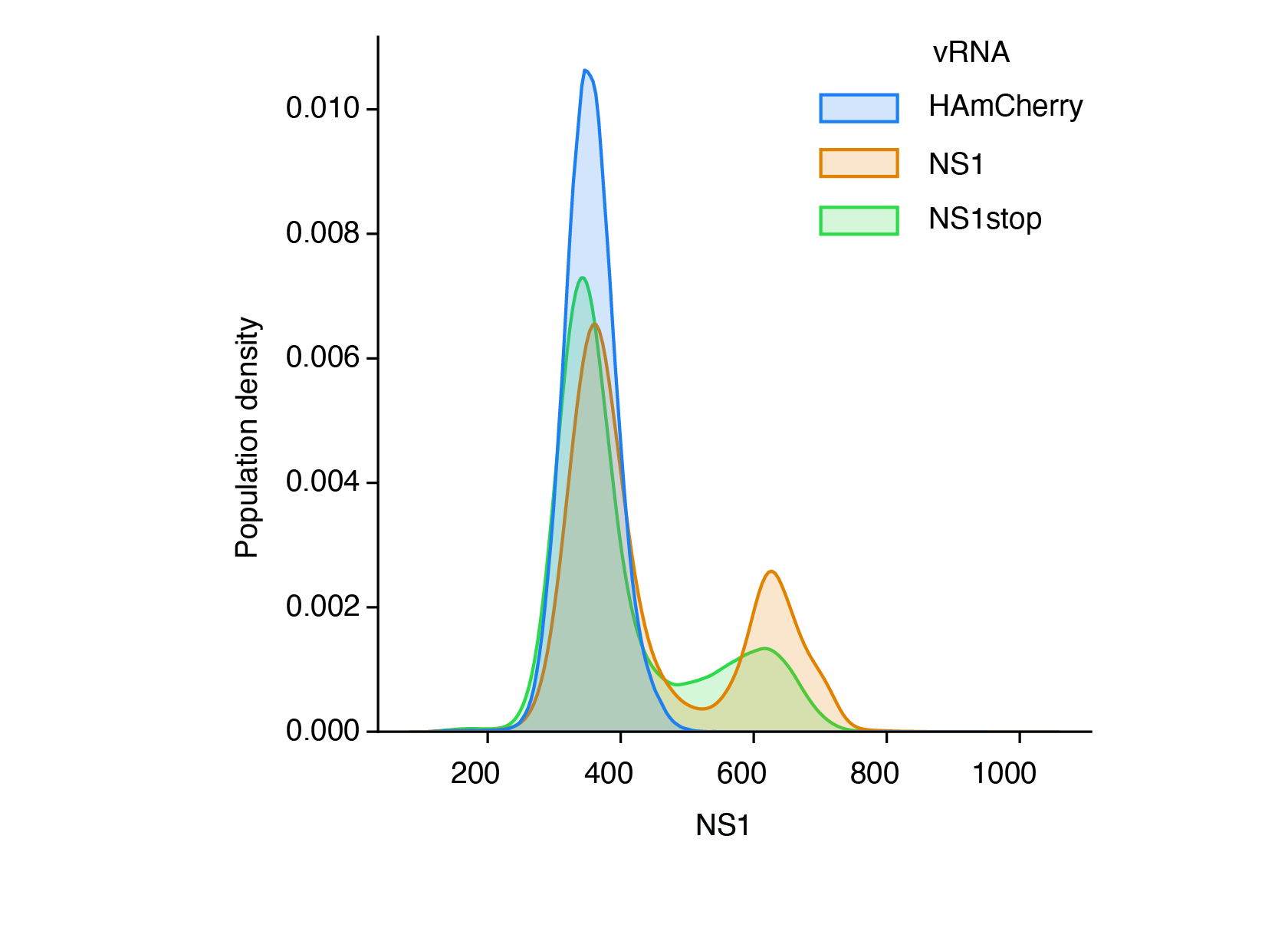

Supplement: S15 Fig — Staining profile of 293T cells transfected with plasmids expressing the four influenza polymerase components (PB2, PB1, PA, and NP) and a plasmid encoding the indicated vRNA. HA-mCherry served as a negative control. (TIF) [file ppat.1010943.s027.tif]

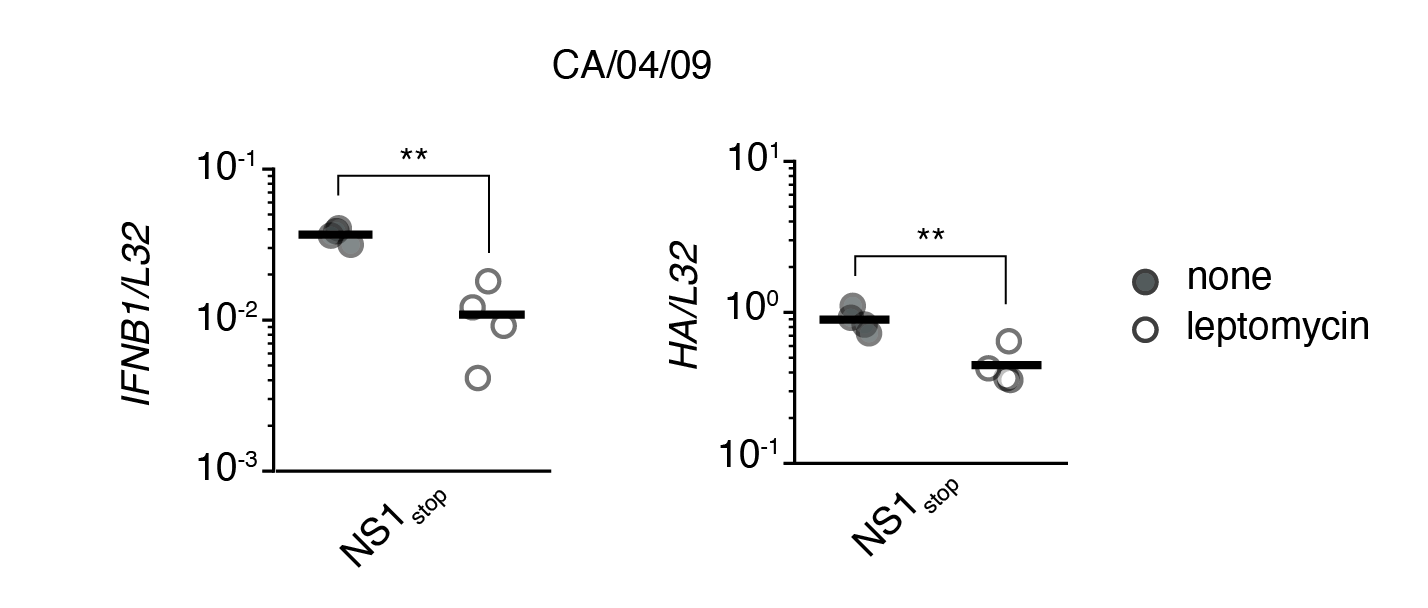

Supplement: S16 Fig — NHBE cells were infected with NS1stop virus at an MOI of 2. At 3 hpi, cells were treated with 10 nM LMB. RNA was harvested at 13.5 hpi, and indicated transcripts analyzed by qPCR against the housekeeping control, L32. Asterisks indicate significant difference, two-tailed t test, p<0.05, n = 4. (TIF) [file ppat.1010943.s028.tif]

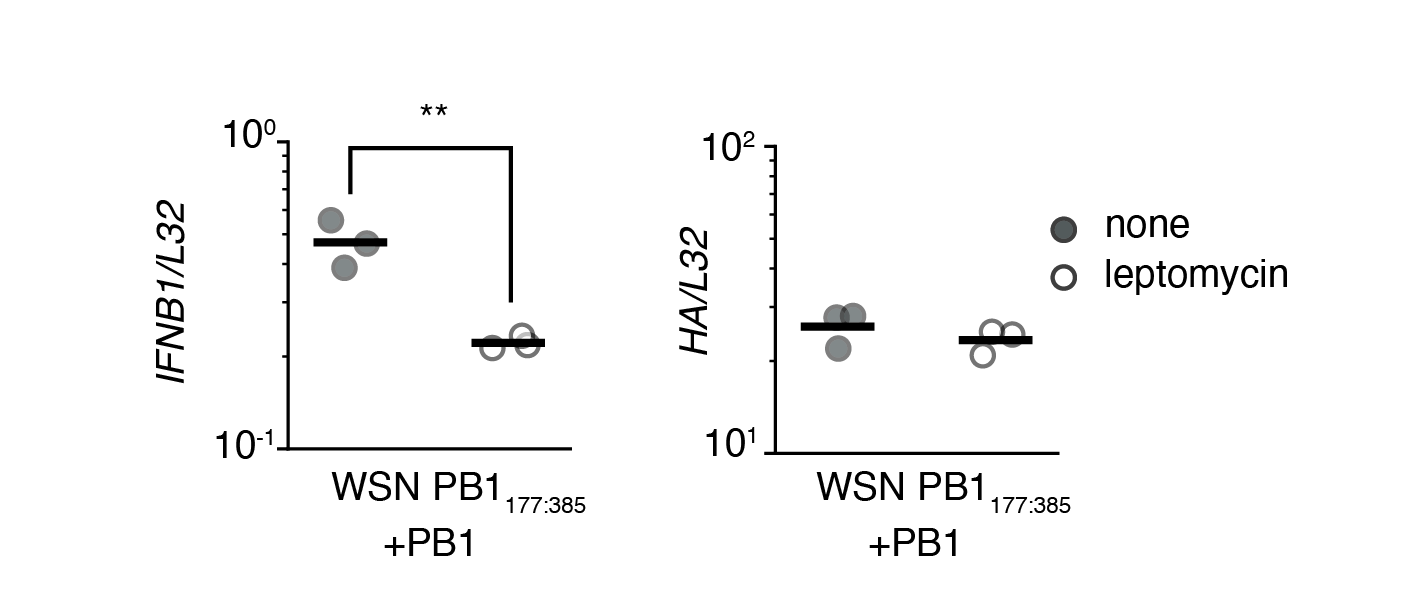

Supplement: S17 Fig — Reporter cells expressing PB1 to permit replication were infected with PB1177:385 virus at an MOI of 0.5. At 3 hpi, cells were treated with 10 nM LMB. This virus was previously shown to be highly stimulatory. [10, 29] RNA was harvested at 8 hpi, and indicated transcripts analyzed by qPCR against the housekeeping control, L32. Asterisks indicate significant difference, two-tailed t test, p<0.05, n = 4. (TIF) [file ppat.1010943.s029.tif]

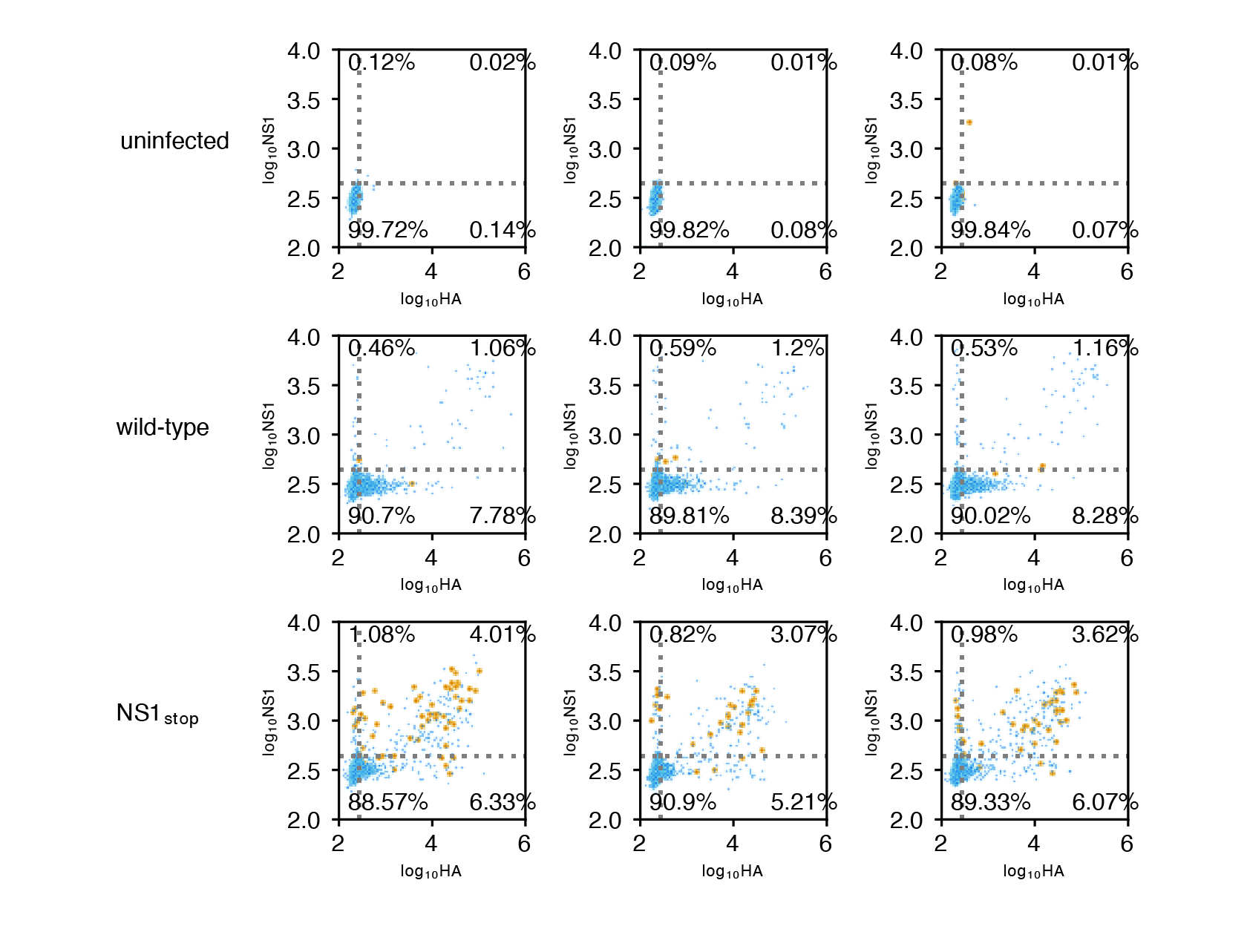

Supplement: S18 Fig — Data showing full flow data and measurements of IFNL1 reporter, HA, and NS1 staining of indicted variants. Cells infected at an MOI of 0.1, measurements made 13h post-infection. Individual replicates shown. Interferon-positive events colored in orange. Data subsetted to 5000 events to show equivalent numbers between conditions. (TIF) [file ppat.1010943.s030.tif]

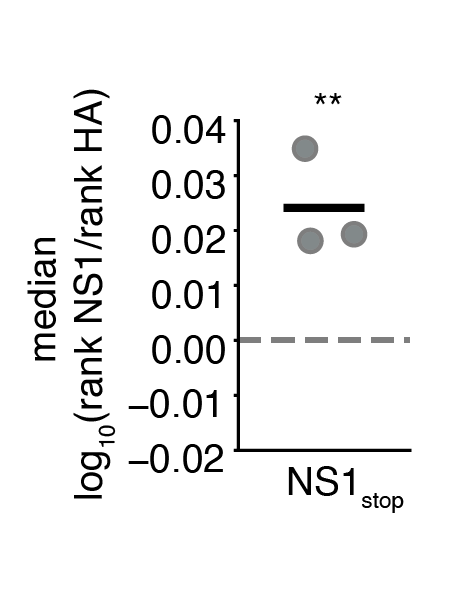

Supplement: S19 Fig — Analysis of NS1stop data from S18 Fig. NS1 and HA staining in double-positive cells were normalized by quantiles, ranking cells by percentile out of one-hundred percent. The NS1 rank in IFNL1+ cells was then divided by the HA rank, if we see less NS1 staining than expected from HA staining the log-transformed value should be less than 0 (dotted line), if more, greater. There is significantly more NS1 staining in IFNL+ cells than would be expected from HA staining, one sample two-tailed t test, p<0.05. (TIF) [file ppat.1010943.s031.tif]

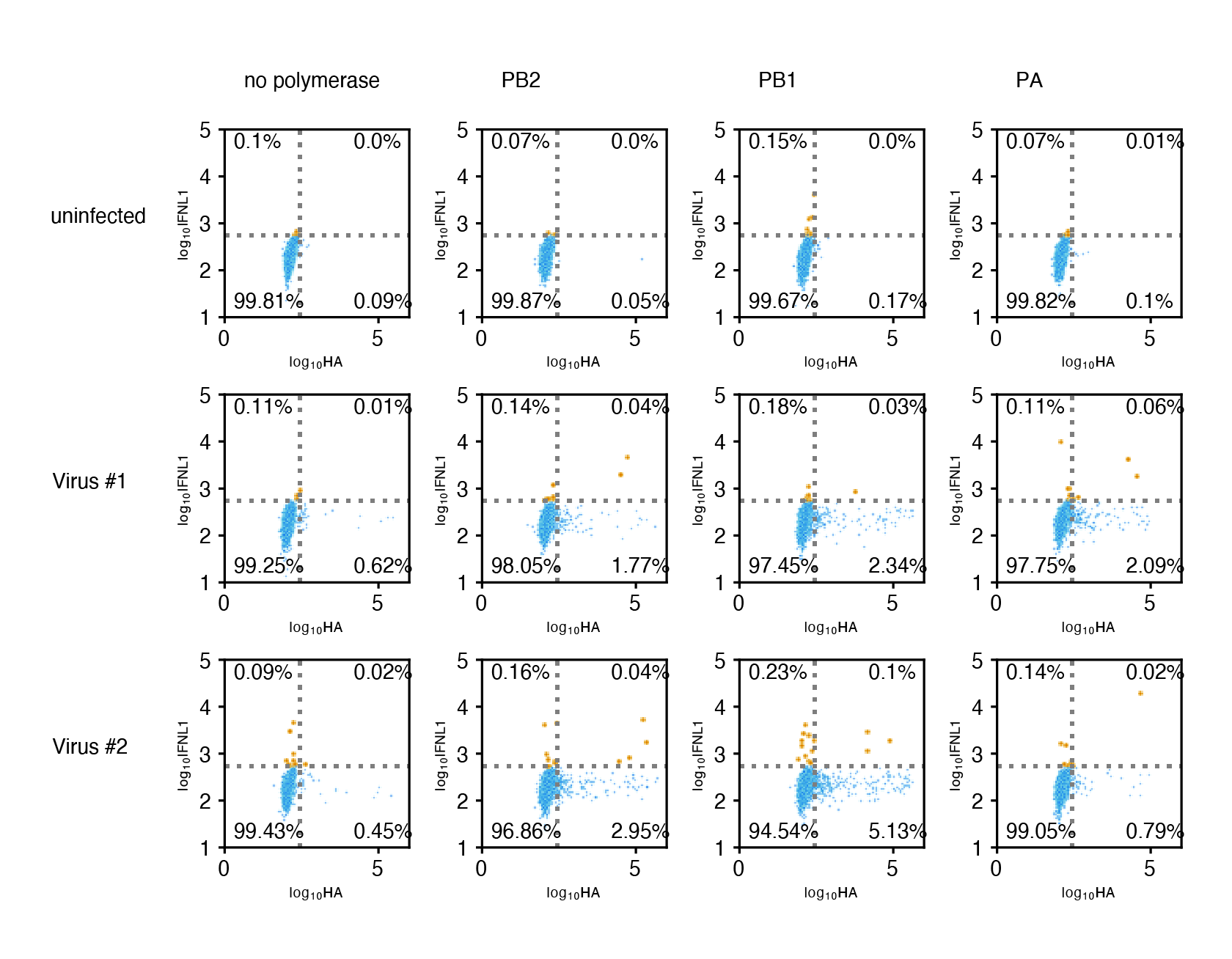

Supplement: S20 Fig — Both high-defective wild-type populations were used to infect IFNL1 reporter cells at an MOI of 0.1, and stained for HA at 13h post infection. IFNL1 reporter cells were complemented, individually, with each of the three polymerase segments. An increase in HA staining was observed in each condition, as well as an increase in the fraction of responding cells. (TIF) [file ppat.1010943.s032.tif]

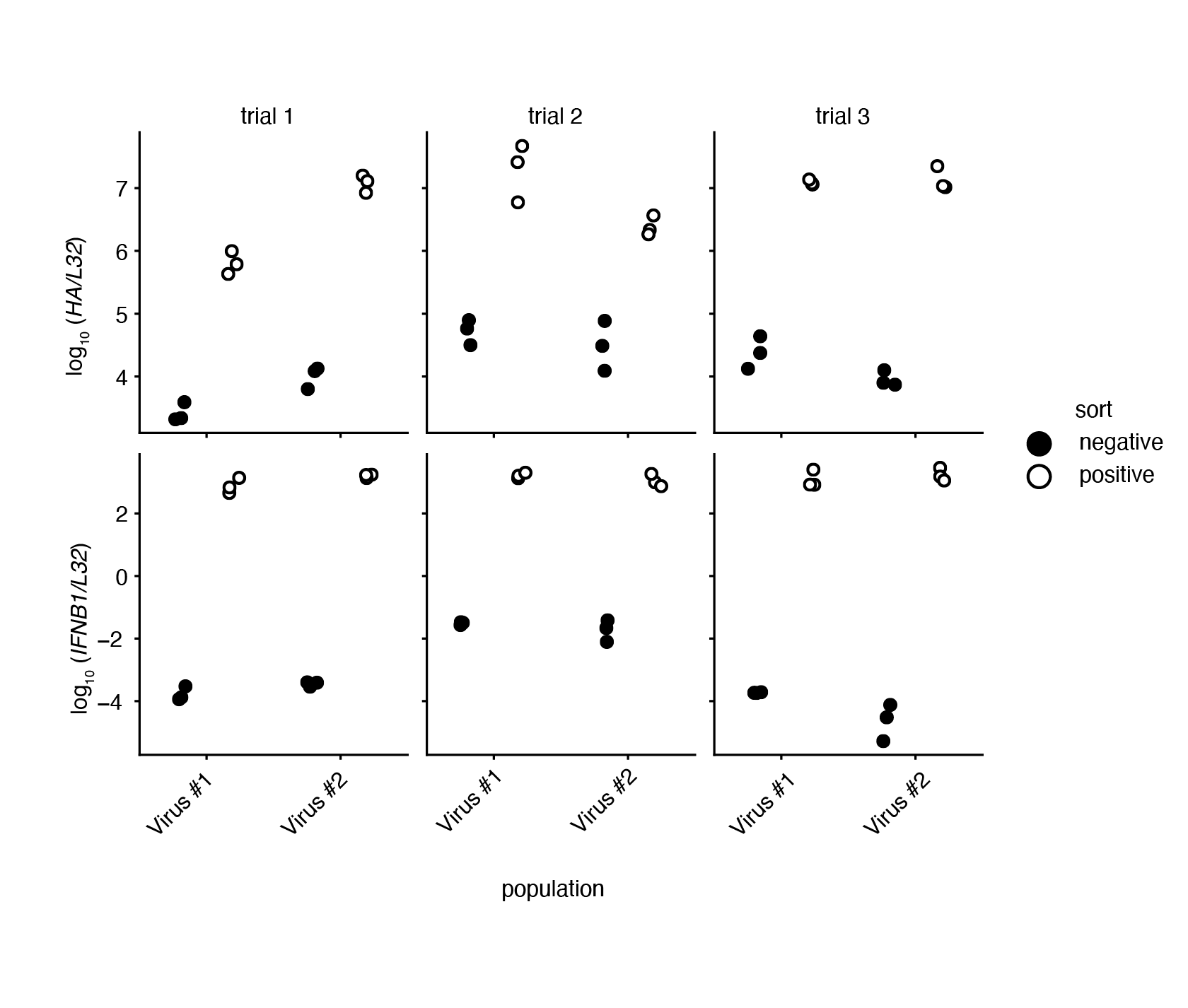

Supplement: S21 Fig — qPCR against HA (top) or IFNβ (bottom) normalized to the housekeeping control L32 of triplicate technical replicate, biological duplicate, high-defective wild-type populations infecting IFNL1 reporter cells, sorted on IFNL1 reporter expression. As expected, both infection and interferon expression was enriched during our sort. Each point indicates a technical replicate measurement of transcript values for each individual experimental technical replicate and biological replicate. (TIF) [file ppat.1010943.s033.tif]

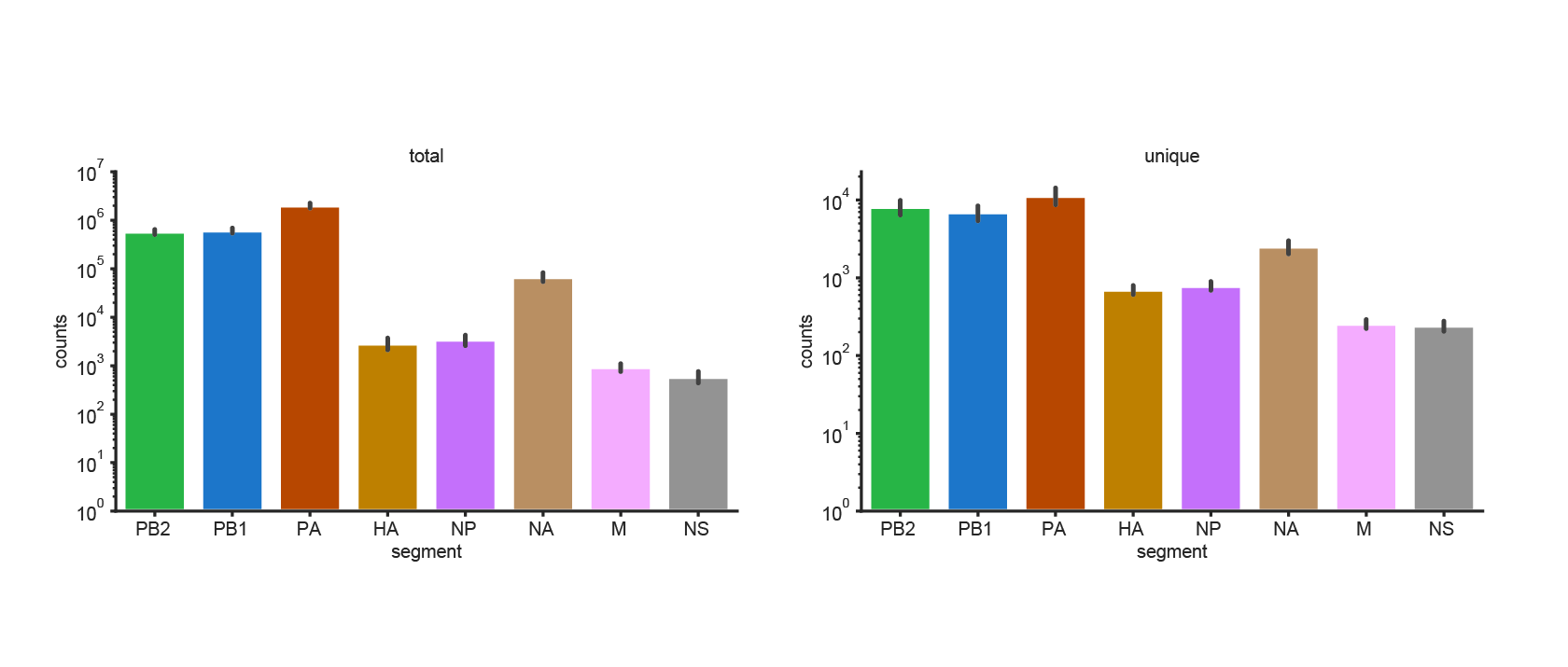

Supplement: S22 Fig — Using the pipeline described in methods, both total fragments, and unique fragments, mapped to deletions in each segment across all six replicate experiments. (TIF) [file ppat.1010943.s034.tif]
